# Enhancing sensitivity in absorption spectroscopy using a scattering cavity

**DOI:** 10.1038/s41598-021-94028-4

**Published:** 2021-07-21

**Authors:** Jeonghun Oh, KyeoReh Lee, YongKeun Park

**Affiliations:** 1 Department of Physics, Korea Advanced Institute of Science and Technology (KAIST), Daejeon, 34141 Republic of Korea; 2 KAIST Institute for Health Science and Technology, Daejeon, 34141 Republic of Korea; 3 Tomocube, Inc., Daejeon, 34051 Republic of Korea

**Keywords:** Optics and photonics, Physics

## Abstract

Absorption spectroscopy is widely used to detect samples with spectral specificity. Here, we propose and demonstrate a method for enhancing the sensitivity of absorption spectroscopy. Exploiting multiple light scattering generated by a boron nitride (h-BN) scattering cavity, the optical path lengths of light inside a diffusive reflective cavity are significantly increased, resulting in more than ten times enhancement of sensitivity in absorption spectroscopy. We demonstrate highly sensitive spectral measurements of low concentrations of malachite green and crystal violet aqueous solutions. Because this method only requires the addition of a scattering cavity to existing absorption spectroscopy, it is expected to enable immediate and widespread applications in various fields, from analytical chemistry to environmental sciences.

## Introduction

Absorption spectroscopy detects a trace of a specific substance by measuring the absorbance of light at various wavelengths^1,2^. Due to its instrumentational simplicity and high spectral specificity, absorption spectroscopy has been exploited in various fields, such as analytical chemistry^3,4^, atmospheric science^5,6^, geology^7^, and atomic physics^8^. The measured absorbance is proportional to the optical path length (OPL) *l* according to the Beer-Lambert law:

1 $$I = I_{0} \exp \,( - A{\kern 1pt} {\kern 1pt} ) = I_{0} \exp \,( - \varepsilon {\kern 1pt} c{\kern 1pt} {\kern 1pt} l{\kern 1pt} {\kern 1pt} )\,,$$

where *I*_0_ and *I* are the measured intensities in the absence of a sample and in the presence of a sample, respectively, *A* is the dimensionless absorbance of the sample, *ε* is the molar attenuation coefficient of the sample, and *c* is the molar concentration of the solution^9^.

The sensitivity of absorption spectroscopy can be enhanced by increasing *l*. For instance, multi-path absorption cells with mirrors have been employed^10–13^. Such systems can significantly amplify the OPL but generally require a bulky system and sophisticated alignment. Attenuated total reflection spectroscopy exploits multiple total internal reflections in a crystal, which creates an evanescent wave^14,15^. However, this method requires a complicated configuration, such as the exact optical contact between a sample and a crystal^16^. The need for a method of using simpler and more compact equipment to increase the OPL has been developed^17^.

Multiple light scattering has been exploited to increase the OPL and overcome the limitations of conventional optical elements^18^, including superlens^19^, temperature^20^, pressure sensing^21^, wavelength meters^22^, fiber-based spectrometers^23^, and non-resonant lasers^24^. The existing approaches employing the multiple scattering regime are represented by an integrating sphere^25–28^. Absorption spectroscopy using an integrating sphere has the advantage of increasing the OPL without the demand for precise alignment of a beam and optical components^25^.

Recently, Martin et al.^29^ suggested multiple scattering for enhancing the sensitivity of absorption spectroscopy. Dielectric microspheres were added to a sample solution to induce multiple scattering, which effectively increased the OPL. However, this method has various limitations: it requires the addition of microspheres for each measurement. Once mixed with microspheres for measurement, the remaining beads prevented the reuse of the sample. In addition, the degree of increase in the OPL varies depending on the quantity and size of the beads, as well as the refractive index (RI) difference between the microspheres and the sample. Thus, the enhancement factor varies according to the experimental environment.

Here, we present a simple but powerful method for increasing the OPL in absorption spectroscopy. By introducing a scattering cavity to existing absorption spectroscopy to enclose a sample, the sensitivity of the measurements is significantly improved, because the light is trapped inside a scattering cavity and interacts with a sample numerous times before exiting. This method does not perturb a sample or require complex instruments. In addition, the method does not require calibration for each measurement and has not interfered with the sample condition. Although there are methods that showed the increase of the OPL using an integrating sphere, our method can be an alternative in the practical perspective because the present scattering cavity is easily combined with an existing commercial cuvette and a spectrometer in the minimal modification. With this regard, we demonstrate that the limit of detection (LOD) can be lowered to what extent with a commercial cuvette.

## Materials and methods

**Figure 1:**
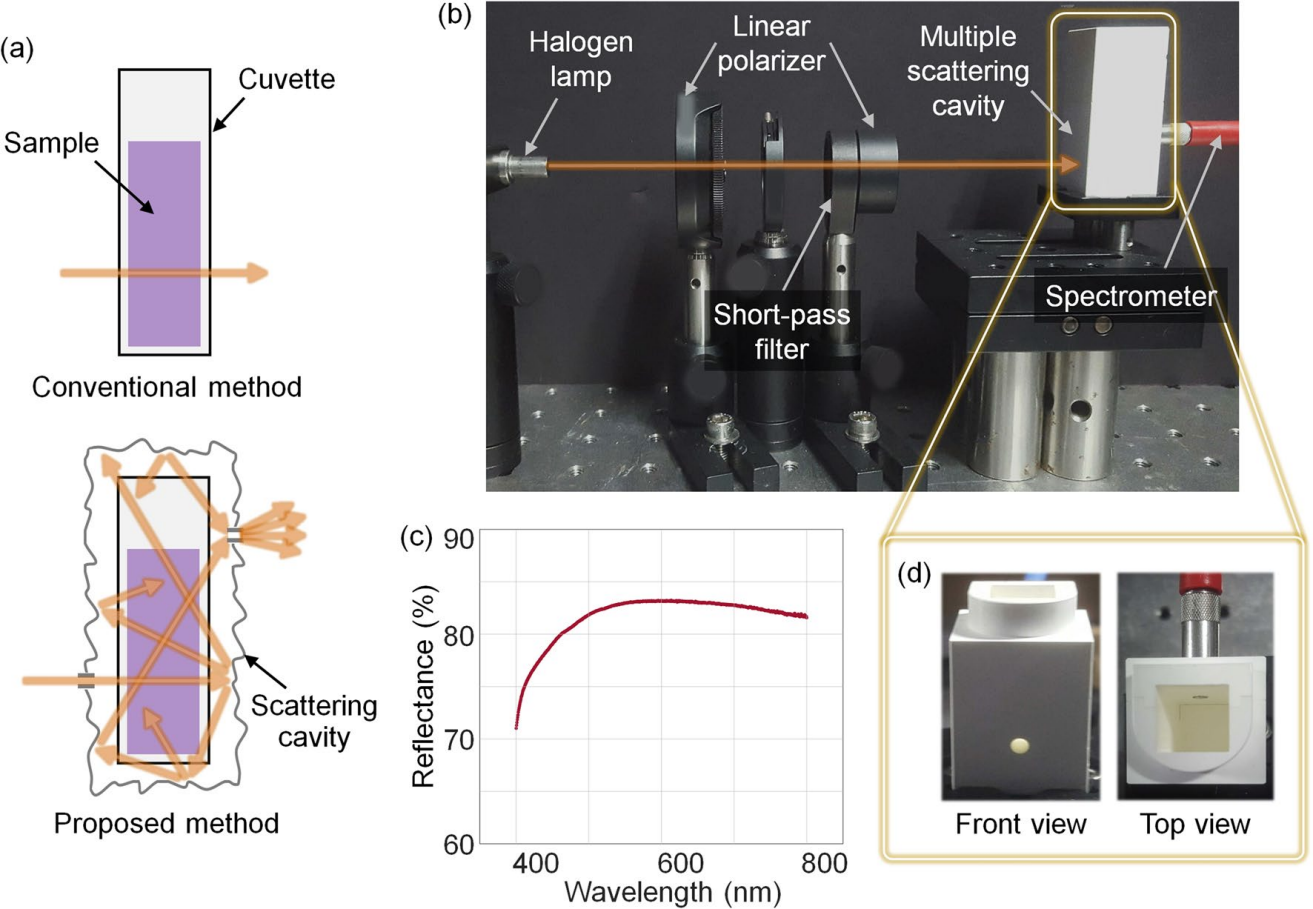
(**a**) Schematics of the conventional method and proposed method. (**b**) Photograph of the experimental setup. (**c**) Reflectance of h-BN. (**d**) Front (left) view and top (right) view of the scattering cavity.

In the conventional method, light passes through a cuvette only once. In the proposed method, the diffusive surface of a scattering cavity scatters light, causing the diffusively reflected light to pass through a longer path, and the reflected light is collected several times (Fig. 1a). The experimental setup was composed of a halogen lamp (OSL1-EC, Thorlabs, Inc.), a custom-made scattering cavity, and a spectrometer (HR4000, Ocean Optics, Inc.) (Fig. 1b). Two linear polarizers (LPVISE100-A, Thorlabs, Inc.) were employed as the beam power attenuators. A short-pass filter (under 750 nm, FES0750, Thorlabs, Inc.) was utilized to filter the wavelengths that exceeded the spectral range of the spectrometer. Note that linear polarizers and short-pass filters are not essential components.

The scattering cavity was made of > 99.5% purity hexagonal boron nitride (h-BN). h-BN exhibits minimal absorbance^30,31^, and high diffuse reflectance in the visible region^32^ makes h-BN suitable for absorption spectroscopy. Due to its graphite-like atomic structure, h-BN exhibits excellent mechanical properties and exhibits satisfactory machinability^33–36^. The diffuse reflectance of h-BN was calibrated using a spectrophotometer (Lambda 1050, Perkin Elmer, Inc.) (Fig. 1c). h-BN has a high diffuse reflectance of more than 80% at wavelengths longer than 500 nm. The inverse adding-doubling method^37,38^ was used to estimate the absorption and scattering coefficients of h-BN. The estimated absorption coefficient $\mu_{a}$ and reduced scattering coefficient $\mu_{s}^{\prime }$ were 0.023 mm^−1^ and 129 mm^−1^, respectively, at 532 nm. The front and top views of the scattering cavity are shown in Fig. 1d. To prevent the direct passing of normal incident light, the exit hole was offset from the entrance with a height difference of 10 mm. The height difference makes the light hit the wall of the scattering cavity and multiply-reflected. Generally, the roughness of the surface will change the specular reflection portion of the reflected light. In our cavity geometry, however, the offset between the entrance and exit holes prevents the domination of the direct or few specular reflections, similar to the function of baffles in a conventional integrating sphere.

## Results

### Measurement of the normalized intensity and absorbance with the scattering cavity

**Figure 2:**
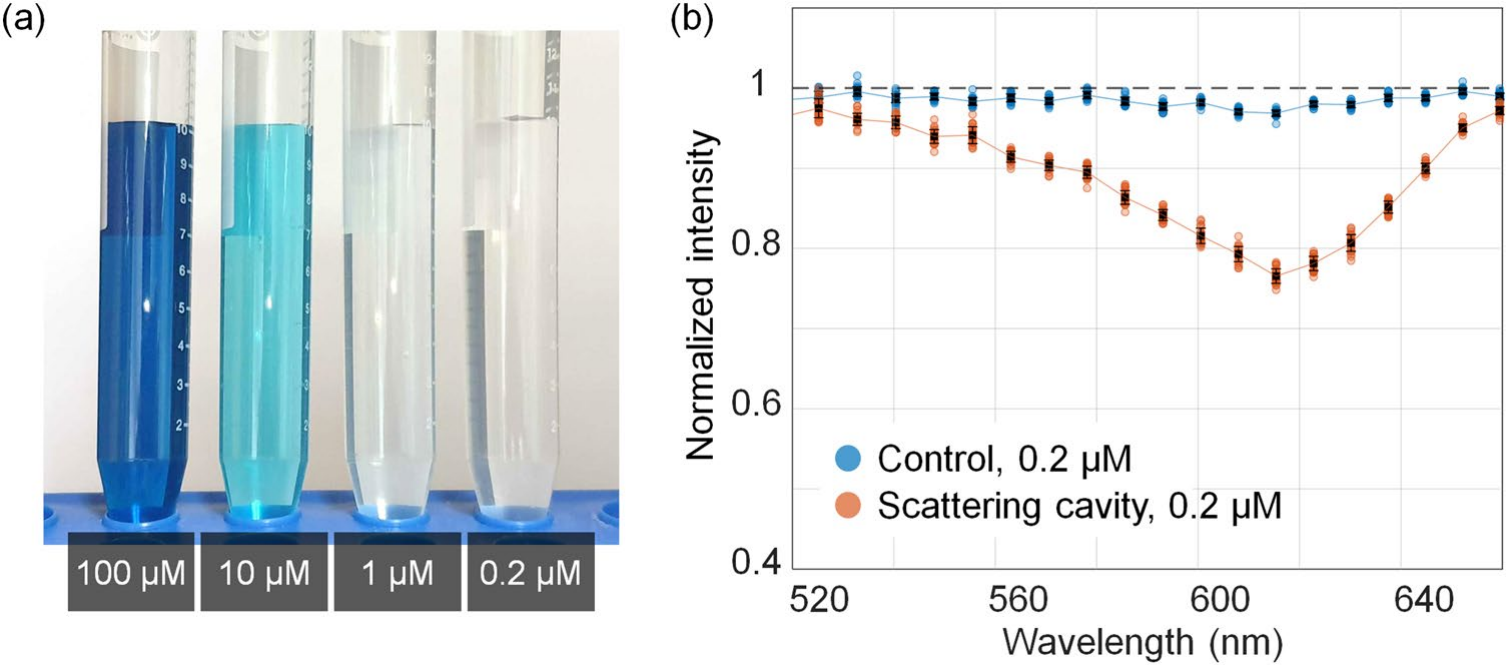
(**a**) Photographs of malachite green aqueous solutions at 100, 10, 1.0, and 0.2 µM. (**b**) Comparison for the normalized absorption spectra of 0.2 µM malachite green aqueous solution measured with the conventional method and the proposed method. Error bars, standard deviations.

To verify this method, the absorption spectra of malachite green and crystal violet were measured. The differentiation of substances from the absorption spectrum of their mixtures has been a difficult task in absorption spectroscopy, especially when two or more substances have overlapped absorption peaks^39^. We demonstrated the proposed method for pure solutions. Malachite green and crystal violet have high water solubility and exhibit maximum absorption at wavelengths of 617 nm and 590 nm, respectively^40,41^. We measured the malachite green aqueous solutions at various concentrations (Fig. 2). It was difficult to discern the malachite green solution with the naked eye when the concentration was below 1 µM (Fig. 2a). The absorption spectra were measured from the sample solution and deionized (DI) water, which correspond to *I* and *I*_0_, respectively, in Eq. (1). The normalized spectrum *I*/*I*_0_ using the conventional method was measured in the same manner. Figure 2b presents the normalized spectra using the proposed and conventional methods for 0.2 µM malachite green aqueous solution. The absorption dip at 617 nm can be clearly observed in the proposed method, whereas the conventional method provides a low signal-to-noise ratio (SNR).

**Figure 3:**
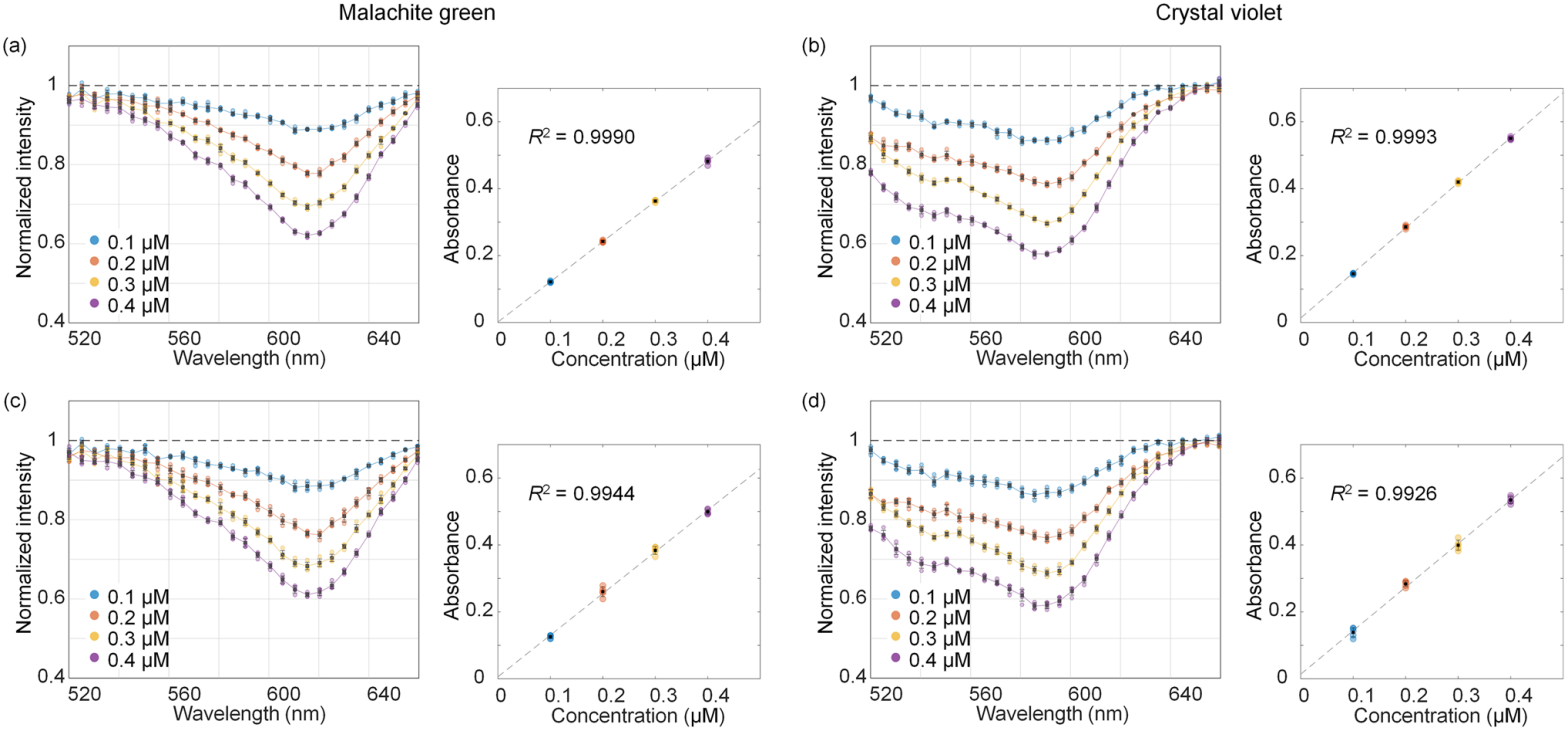
Normalized spectra and absorbance at the dip wavelength at the concentrations of 0.1, 0.2, 0.3, and 0.4 µM for (**a**, **c**) malachite green and (**b**, **d**) crystal violet aqueous solutions. The absorbance at the dip wavelength as a function of concentrations is represented together. (**a**, **b**) are the results of measuring five times for the same solution; (**c**, **d**) are the results of producing five solutions and measuring for each solution. The black dots represent the average of the results conducted five times. Error bars, standard deviations.

The normalized spectra of malachite green and crystal violet aqueous solutions at various concentrations are shown in Fig. 3. For validation, five measurements were acquired from the same sample solution (Fig. 3a,b) and five individual solutions of the same concentration (Fig. 3c,d). We discovered that the absorption dips were consistently observed at 617 nm and 590 nm for malachite green and crystal violet, respectively, which shows an agreement with the known values. In addition, the linear relationship between concentration and absorbance in Eq. (1) is shown with high R-square values in the graphs of the absorbance (Fig. 3a–d). The linearity of the Beer-Lambert law with the effective OPL is valid for the solutions of low concentrations^25^. These results show the robustness of the presented method using multiple scattering.

### Enhancement factor for the sensitivity of detection

**Figure 4:**
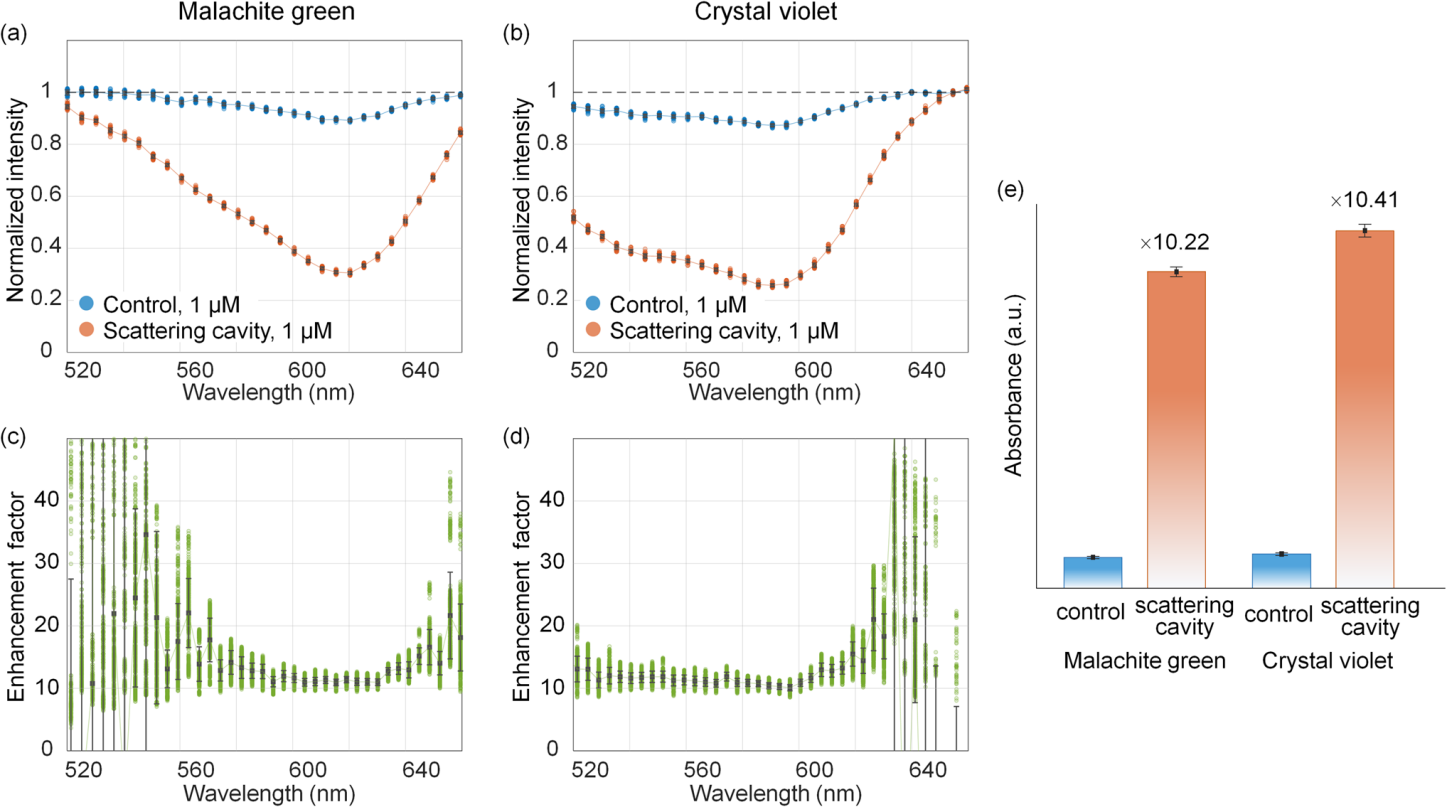
Normalized intensity depending on the wavelength at the concentration of 1 µM for (**a**) malachite green and (**b**) crystal violet aqueous solutions using a conventional method and the scattering cavity. At each wavelength, 25 measurements were performed. The enhancement factors as a function of the wavelength for (**c**) malachite green and (**d**) crystal violet aqueous solutions. (**e**) The averaged enhancement factors near a dip of the normalized intensity for malachite green and crystal violet aqueous solutions. Error bars, standard deviations.

According to the results using the scattering cavity in Fig. 3, it is possible to see a distinct dip of the normalized intensity. To quantify the enhancement in absorbance (−log(*I*/*I*_0_)) of the proposed method, we calculated the ratio of the absorbances between the proposed method and the conventional method (control) using the same sample solution (malachite green and crystal violet, 1 µM) (Fig. 4a,b). The normalized intensity depending on the wavelength contains 25 data for each case. Figure 4c,d show the enhancement factor as a function of the wavelength for both aqueous solutions. For wavelengths near the dips, the enhancement factors were almost constant regardless of the wavelength. The highly variable enhancement in the low-absorbance regimes is due to the near-zero absorbance of the control experiments. The averaged enhancement factors at the wavelengths near the dip were 10.22 times and 10.41 times for malachite green and crystal violet, respectively (Fig. 4e). Because the increment in the OPL is not dependent on the sample, the result of a similar enhancement for the two samples is reasonable.

### Limit of detection

**Figure 5:**
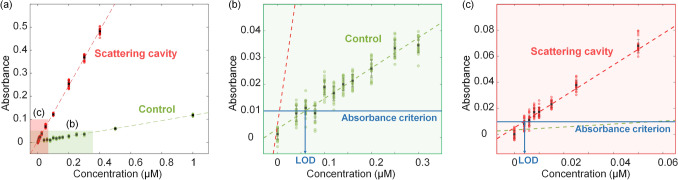
(**a**) Absorbance in malachite green aqueous solutions of low concentrations measured by the conventional method (green) and the scattering cavity (red). (**b**, **c**) Enlarged shaded areas. The measurements were made 25 times each for all concentrations. Error bars, standard deviations. The absorbance criterion (*μ*_0_ + 3*σ*_0_) for estimating the concentration corresponding to the LOD is marked with a blue line.

The LOD is the lowest concentration of a sample that discernable with the absence of the sample^42^ and can be lower by enhancing the measured absorbance. To determine the LOD of the proposed system, we used highly diluted malachite green solutions (Fig. 5). The LOD was obtained by interpolating the concentration that yields the absorbance criterion *μ*_0_ + 3*σ*_0_, where *μ*_0_ and *σ*_0_ are the mean and standard deviation, respectively, of the results using pure water (0 µM) ^42,43^. The evaluation of the LOD via pure water (blank solution) fundamentally reflects the systematic error of the detection system.

Figure 5a shows the absorbance measurements for the LOD calibration. We employed malachite green solutions at concentrations of 0.040 µM and 0.004 µM for the conventional system (control) and proposed system, respectively. Figure 5b,c show the shaded regions in Fig. 5a. The absorbance was calculated from the mean and standard deviation of the 0 µM (pure water) absorbance results. We found that the absorbance criteria were *μ*_0_ + 3*σ*_0_ = 0.01 in both cases. Note that the absorbance criteria should be the same in both cases because the zero fluctuations are solely derived from the sensitivity and precision of the detection system. In the conventional method (control), the linear correlation was maintained up to 0.120 µM, but the deviation increased for concentrations lower than 0.100 µM (Fig. 5b). The calibrated LOD was 0.059 µM, which was consistent with the data observations. In the proposed method, it is noteworthy that the linearity was maintained at substantially lower concentrations (minimum of 0.010 µM) than in the conventional method (Fig. 5c). The calibrated LOD was 0.004 µM, which is lower than 1/10 of the LOD of the control.

## Conclusion

In summary, we present a scattering cavity to enhance the sensitivity of absorption spectroscopy via a significant increase in the OPL. The scattering cavity was composed of h-BN and led to diffusive reflection on its surface, achieving an improvement in the absorbance by more than 10 times. Moreover, the LOD was drastically lowered in the scattering cavity, and malachite green could be detected even at 0.004 µM using a commercial cuvette. We expect that a higher diffuse reflectance will further improve the enhancement factor. For example, a commercially available highly reflective surface shows > 99% diffuse reflectance in the visible and near-infrared range^44^; many common ceramics (such as alumina and zirconia) or synthetic polymers (such as PTFE) with high reflectance can be utilized instead. Furthermore, the proposed method may be improved by optimizing the size of the entrance and the scattering cavity for further enhancement of the OPL^24^.

It should be emphasized that the proposed method using multiple scattering is more robust against misalignment than the methods using specular reflection of a mirror. In addition, the proposed method enables quick and simple measurement of absorbance using a plastic cuvette, which is commonly employed in analytical chemistry and can be readily applicable to existing spectrometers. Due to the effectiveness of the proposed method in detecting trace amounts of a sample mixed in a liquid solvent, the method is expected to widen the detection range for a substance and the wavelength of interest, expanding the research area. We believe that our system can be beneficial for practical applications that require low LOD in water^45^, such as those in the food industry^46^, pathological diagnosis^47^, and biochemical sensing^48,49^.

## References

[1] McManus JB, Kebabian PL, Zahniser M (1995). Astigmatic mirror multipass absorption cells for long-path-length spectroscopy. Appl. Opt..

[2] Platt, U. & Stutz, J. Differential absorption spectroscopy. in *Differential optical absorption spectroscopy* 135–174 (Springer, 2008).

[3] BV, D.C. & Hydraulics, D. Absorption spectroscopy. (1962).

[4] Fuller M (1997). Chemical characterization of tribochemical and thermal films generated from neutral and basic ZDDPs using X-ray absorption spectroscopy. Tribol. Int..

[5] Brown SS (2003). Absorption spectroscopy in high-finesse cavities for atmospheric studies. Chem. Rev..

[6] Werle P, Mücke R, Slemr F (1993). The limits of signal averaging in atmospheric trace-gas monitoring by tunable diode-laser absorption spectroscopy (TDLAS). Appl. Phys. B.

[7] Chen Y (2015). Applications of micro-fourier transform infrared spectroscopy (FTIR) in the geological sciences: A review. Int. J. Mol. Sci..

[8] Van Loon AT (2012). Analytical atomic absorption spectroscopy: Selected methods.

[9] Dean JA, Dean J (1995). Analytical chemistry handbook.

[10] White JU (1942). Long optical paths of large aperture. JOSA.

[11] Herriott DR, Schulte HJ (1965). Folded optical delay lines. Appl. Opt..

[12] Romanini D, Kachanov A, Sadeghi N, Stoeckel F (1997). CW cavity ring down spectroscopy. Chem. Phys. Lett..

[13] Robert C (2007). Simple, stable, and compact multiple-reflection optical cell for very long optical paths. Appl. Opt..

[14] Averett LA, Griffiths PR, Nishikida K (2008). Effective path length in attenuated total reflection spectroscopy. Anal. Chem..

[15] Fahrenfort J (1961). Attenuated total reflection: A new principle for the production of useful infra-red reflection spectra of organic compounds. Spectrochim. Acta.

[16] Grdadolnik J (2002). ATR-FTIR spectroscopy: Its advantage and limitations. Acta Chim. Slov..

[17] Tuzson B, Mangold M, Looser H, Manninen A, Emmenegger L (2013). Compact multipass optical cell for laser spectroscopy. Opt. Lett..

[18] Park JH, Park J, Lee K, Park Y (2020). Disordered optics: Exploiting multiple light scattering and wavefront shaping for nonconventional optical elements. Adv. Mater..

[19] Park J-H (2013). Subwavelength light focusing using random nanoparticles. Nat. Photon..

[20] Trivedi V (2014). Optical temperature sensor using speckle field. Sens. Actuat. A.

[21] Kim K (2016). Remote sensing of pressure inside deformable microchannels using light scattering in Scotch tape. Opt. Lett..

[22] Mazilu M, Vettenburg T, Di Falco A, Dholakia K (2014). Random super-prism wavelength meter. Opt. Lett..

[23] Redding B, Cao H (2012). Using a multimode fiber as a high-resolution, low-loss spectrometer. Opt. Lett..

[24] Lee K, Ma HJ, Rotermund F, Kim DK, Park Y (2021). Non-resonant power-efficient directional Nd: YAG ceramic laser using a scattering cavity. Nat. Commun..

[25] Hodgkinson J, Masiyano D, Tatam RP (2009). Using integrating spheres as absorption cells: Path-length distribution and application of Beer's law. Appl. Opt..

[26] Pope RM, Fry ES (1997). Absorption spectrum (380–700 nm) of pure water II. Integrating cavity measurements. Appl. Opt..

[27] Villanueva Y, Veenstra C, Steenbergen W (2016). Measuring absorption coefficient of scattering liquids using a tube inside an integrating sphere. Appl. Opt..

[28] Elterman P (1970). Integrating cavity spectroscopy. Appl. Opt..

[29] Koman VB, Santschi C, Martin OJ (2015). Multiscattering-enhanced absorption spectroscopy. Anal. Chem..

[30] Watanabe K, Taniguchi T, Kanda H (2004). Direct-bandgap properties and evidence for ultraviolet lasing of hexagonal boron nitride single crystal. Nat. Mater..

[31] Wang J, Ma F, Sun M (2017). Graphene, hexagonal boron nitride, and their heterostructures: properties and applications. RSC Adv..

[32] Spiridonov, D., Henaish, A., Vokhmintsev, A. & Weinstein, I. Diffuse reflectance spectral features of hexagonal boron nitride nanopowder. in *AIP conference proceedings*, Vol. 1886 020021 (AIP Publishing LLC, 2017).

[33] Ooi N, Rajan V, Gottlieb J, Catherine Y, Adams J (2006). Structural properties of hexagonal boron nitride. Modell. Simul. Mater. Sci. Eng..

[34] Eichler J, Lesniak C (2008). Boron nitride (BN) and BN composites for high-temperature applications. J. Eur. Ceram. Soc..

[35] Zunger A, Katzir A, Halperin A (1976). Optical properties of hexagonal boron nitride. Phys. Rev. B.

[36] Song L (2010). Large scale growth and characterization of atomic hexagonal boron nitride layers. Nano Lett..

[37] Prahl SA, van Gemert MJ, Welch AJ (1993). Determining the optical properties of turbid media by using the adding–doubling method. Appl. Opt..

[38] Prahl S (2011). Everything I think you should know about Inverse Adding-Doubling.

[39] Belal TS, Daabees HG, Abdel-Khalek MM, Mahrous MS, Khamis MM (2013). New simple spectrophotometric method for determination of the binary mixtures (atorvastatin calcium and ezetimibe; candesartan cilexetil and hydrochlorothiazide) in tablets. J. Pharm. Anal..

[40] Modirshahla N, Behnajady MA (2006). Photooxidative degradation of Malachite Green (MG) by UV/H2O2: Influence of operational parameters and kinetic modeling. Dyes Pigm..

[41] Gupta A, Pal A, Sahoo C (2006). Photocatalytic degradation of a mixture of Crystal Violet (Basic Violet 3) and Methyl Red dye in aqueous suspensions using Ag+ doped TiO2. Dyes Pigm..

[42] Long GL, Winefordner JD (1983). Limit of detection. A closer look at the IUPAC definition. Anal. Chem..

[43] Shrivastava A, Gupta VB (2011). Methods for the determination of limit of detection and limit of quantitation of the analytical methods. Chronic Young Sci..

[44] Kokaly RF, Skidmore AK (2015). Plant phenolics and absorption features in vegetation reflectance spectra near 1.66 μm. Int. J. Appl. Earth Observ. Geoinform..

[45] Heberer T (2002). Tracking persistent pharmaceutical residues from municipal sewage to drinking water. J. Hydrol..

[46] Terry LA, White SF, Tigwell LJ (2005). The application of biosensors to fresh produce and the wider food industry. J. Agric. Food Chem..

[47] Bieschke J (2000). Ultrasensitive detection of pathological prion protein aggregates by dual-color scanning for intensely fluorescent targets. Proc. Natl. Acad. Sci..

[48] Slack JA (2005). Biochemical markers of cardiac injury in normal, surviving septic, or nonsurviving septic neonatal foals. J. Vet. Intern. Med..

[49] Caucheteur C, Guo T, Albert J (2015). Review of plasmonic fiber optic biochemical sensors: Improving the limit of detection. Anal. Bioanal. Chem..

